# Effects of different intensities of intermittent pneumatic soft-tissue compression on bone defect repair

**DOI:** 10.1186/s12891-022-05341-6

**Published:** 2022-04-30

**Authors:** Weilong Diwu, Gang Hu, Minghao Zhou, Long Bi, Ming Yan, Hongbo Wei, Junjun Fan

**Affiliations:** 1 Department of Orthopedics, The First Affiliated Hospital, The Fourth Military Medical University, Xi’an, China; 2 Department of Oral Implants, School of Stomatology, State Key Laboratory of Military Stomatology, National Clinical Research Center for Oral Diseases & Shaanxi Engineering Research Center for Dental Materials and Advanced Manufacture, The Fourth Military Medical University, Xi’an, China

**Keywords:** Intermittent pneumatic soft-tissue compression, Bone defect, Intensity, Bone repair, Orthopedic

## Abstract

**Background:**

To estimate the effects of different intensities of intermittent pneumatic soft-tissue compression on bone defect repair in an animal model.

**Methods:**

Five mm radial bone defect in length was made in 64 mature New Zealand rabbits and all animals randomly assigned into four groups: Group A (control group without compression), Group B (5–7 kPa intensity), Group C (8–10 kPa intensity) and Group D (11–13 kPa intensity). On the fourth day after surgery, their legs were intermittently pneumatic compressed for 4 weeks. The stimulation lasted 30 min every day and the frequency of compression was 15 Hz. New bone formation in 4 groups was evaluated by gross observation, X-ray, Micro-CT, and histological staining at 2 and 4 weeks after surgery.

**Result:**

There was more new bony callus in the bone defect in group C than in other groups by gross observation and X-ray radiography at 2 and 4 weeks. The Micro-CT results showed more new bony callus, bone trabecula and higher bone mineral density in group C. Fluorescent labeling results showed the speed of new bone formation in Group C was faster than that in other groups, among which the control group had the slowest speed of new bone formation. The result of histology had shown that the trabeculae in bone callus in group C had a regular form, the trabeculae were wide and had a more become osteoblast around them.

**Conclusion:**

The intermittent pneumatic soft-tissue compression can accelerate new bone formation of bone defects and the optimal intensity is 8–10 kPa for repairing the rabbit radial bone defect.

## Introduction

Bone defect treatment aims to achieve osteogenesis and vascularization as soon as possible. This has led to the introduction of several treatment modalities to enhance healing and expedite recovery [1]. Intermittent pneumatic compression (IPC) is a special technique that has been mainly used in orthopedic practice to decrease the risk of deep vein thrombosis (DVT) and to reduce post-traumatic and post-operative swelling in various clinical situations [2–4]. Physicians have tried to improve circulation by exerting external pressure on the arms and legs since nineteenth century. IPC can provide the cyclic positive and negative pressure and improve the arterial circulation [5, 6]. Cyclic pressure of IPC on muscles and blood vessels can decrease the venous stasis and increase the arterial blood flow. IPC cyclic compression on blood vessels leads to the increase of the synthesis of nitric oxide, prostacyclin, and tissue plasminogen activator. Meanwhile, the plasminogen activator inhibitor decreased [7, 8]. Blood circulation is recognized as an important factor in fracture-healing. Therefore, an increase in arterial blood flow to the fracture site may improve healing. With evidence that IPC can enhance bone fractures with good functional recovery, IPC has been approved for clinical treatment on delayed union or nonunion bone fractures. In previous studies, IPC has been proved to be effective for bone fracture healing by enhancing osteogenesis and vascularization [9–12]. But until now, no study has investigated the effect of the optimal intensity of IPC on bone defects. To guide the clinical application, the current study was designed to determine if the beneficial effects of IPC were affected by the treatment intensity.

## Materials and methods

### Animal model and surgery

All experimental procedures were reviewed and approved by the Committee of Ethics on Animal Experiments of the Air Force Military Medical University. A total of 64 adult male New Zealand white rabbits underwent standard radius osteotomy in the right foreleg according to the previous protocol [13]. Each animal was anesthetized with intramuscular injection of ketamine (35 mg/kg), and the skin of the right foreleg was incised. Blunt dissection was carried down to the level of the periosteum. The periosteum was incised, and a 5 mm bone segment in the middle of radius was resected using a wire saw to get a bone defect. The periosteum was then re-approximated, and the wound was closed in layers. Antimicrobial therapy was provided with penicillin for 3 days. After 2 and 4 weeks using intermittently pneumatic compressed, 8 animals from each group were humanely killed by an overdose intravenous injection of pentobarbital (25 mg/kg) after the last intermittent pneumatic compression. After euthanasia, radius containing the bone defect and its associated ulna were removed and examined by X-ray, Micro-computerized tomography (Micro-CT), and histological evaluations.

### Intermittent pneumatic compression

A self-developed pneumatic tourniquet was applied around the upper right foreleg of each rabbit proximal to the defect site. All 64 animals were divided randomly into four groups consisting of 16 rabbits in each group: Group A (control group without compression), Group B (5–7 kPa intensity), Group C (8–10 kPa intensity) and Group D (11–13 kPa intensity). On the fourth day after osteotomy surgery, their legs were intermittently pneumatic compressed for 2 or 4 weeks. The stimulation lasted 30 min every day and the frequency of compression was 15 Hz. The control animals were equipped with identical but nonfunctional tourniquets.

### X-ray

At the second and the fourth week after the surgery, radiograph images were undertaken and each radiograph was examined by two independent observers and given a score depending on a standardized scoring system according to the lane-sandhu scoring standards [14]. Then animals were euthanized. All bone defects and surrounding tissue were taken out for follow-up testing.

### Micro-CT

The microarchitecture of the newly formed bone at bone defects was evaluated by Micro-CT (mCT 40; Scanco Medical). The samples were prepared according to the commonly used procedure [15]. In brief, the specimens were carefully dissected and fixed in 4% neutral buffered formalin and then washed with 0.9% saline to remove the residual formalin. Each specimen was placed in the sample holder with their long axes in the vertical position and scanned using the conditions of 70 kV, 114 mA, and 30 mm voxel size. Scanning data of each specimen were then processed with a 3-dimensional (3D) Gaussian filter and a global threshold to extract bone from soft tissue or bone marrow for subsequent analysis. Morphological parameters of the newly formed bone, such as trabecular number (Tb.N), trabecular spacing (Tb.Sp), and bone mineral density (BMD), were calculated.

### Fluorochrome labeling

Fluorochrome Labeling test was performed according to the commonly used procedure [16]. Sequential fluorochrome markers Calcein (30 mg/kg, SigmaAldrich) and Tetracycline (30 mg/kg, Sigma-Aldrich) were administered via intramuscular injection 2 weeks and 4 days before the animals were humanely killed, respectively. Four weeks post-surgery, animals were humanely killed and pathological sections were applied for fluorescence analysis using an epifluorescence microscopy. The speed of new bone formation was measured by the length between two labels over time (10 days).

### Histology analysis

After two and four weeks, histology analysis samples were prepared according to the commonly used procedure [16].

### Statistical analysis

All statistical analyses were performed using SPSS 17.0 software (SPSS Inc). All quantitative data were expressed and reported as the mean and the standard deviation. Levels were compared by the one-way ANOVA and Student’s t-test. P values less than 0.05 were considered significant.

## Results

### X-ray analyses

**Fig. 1:**
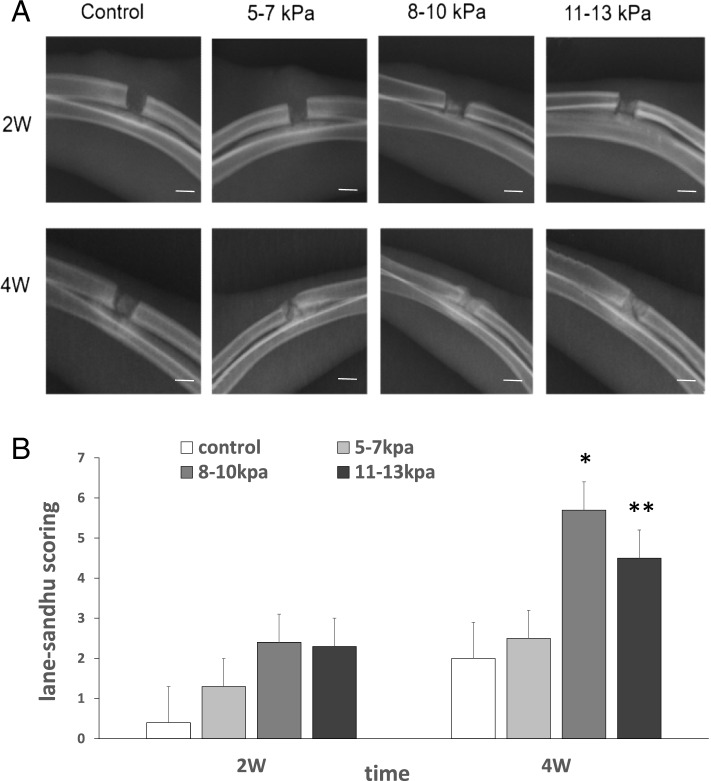
X-rays of bone defects 2 and 4 weeks after surgery. Notes: **A** X-rays of bone defects to visualize healing of bone defects in the rabbit radius at 2 and 4 weeks. scale bars: 5 mm. **B** The lane-sandhu scoring values were compared in different groups. Results are mean ± standard deviation (χ ± SD); *P < 0.05 vs group **A**,**B**,**D**; **P < 0.05 vs group **A**,**B**

X-rays of bone defects at second and fourth weeks after implantation were analyzed (Fig. 1). Two weeks after implantation, the bone defect in groups A and B were nearly invisible because of the low X-ray attenuation coefficient. In groups C and D, new bone tissue appeared at the radial defect sites. Four weeks after implantation, massive new bone tissue appeared and filled the bone defect sites in groups A, B, and C, among which less new bone tissue appeared in group A. According to the lane-sandhu scoring standards, group C had the highest score in all groups.

### Micro-CT results

**Fig. 2:**
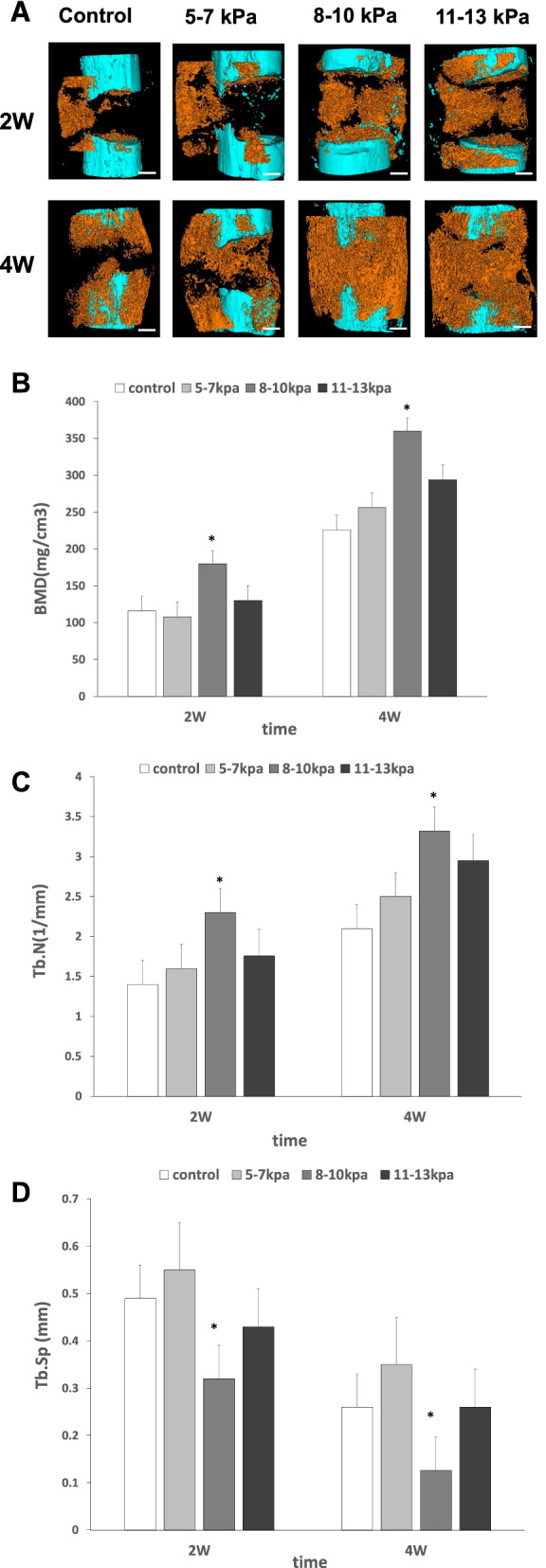
Micro-CT of bone defects 2 and 4 weeks after surgery. Notes: **A** Micro-CT scans and 3D reconstructions to visualize healing of bone defects in the rabbit radius at 2 and 4 weeks. The newly formed radius bone is shown in orange in the Micro-CT images; scale bars: 2 mm. **B** Bone mineral density values. **C** Trabecular number (Tb.N) values. **D** Trabecular spacing (Tb.Sp) values. Results are mean ± standard deviation (χ ± SD); *P < 0.05 vs group **A**, **B**, **D**

Micro-CT was used to demonstrate 3D images of the bone defect area as well as to determine the quantity of newly formed bone (Fig. 2). After 2 weeks, new bone tissue was mainly around the defect sites and invisible inside the defect in groups A and B. More newly formed bone appeared inside the defect sites in groups C and D. Group C had the highest bone mineral density (BMD), trabecular number (Tb.N), and least trabecular spacing (Tb.Sp) compared with other groups. After 4 weeks, newly formed bone increased in all groups but only groups C and D had massive new bone tissue filled the defects. Group C still had the highest bone mineral density, trabecular number, and least trabecular spacing compared with other groups.

### Fluorochrome labeling

**Fig. 3:**
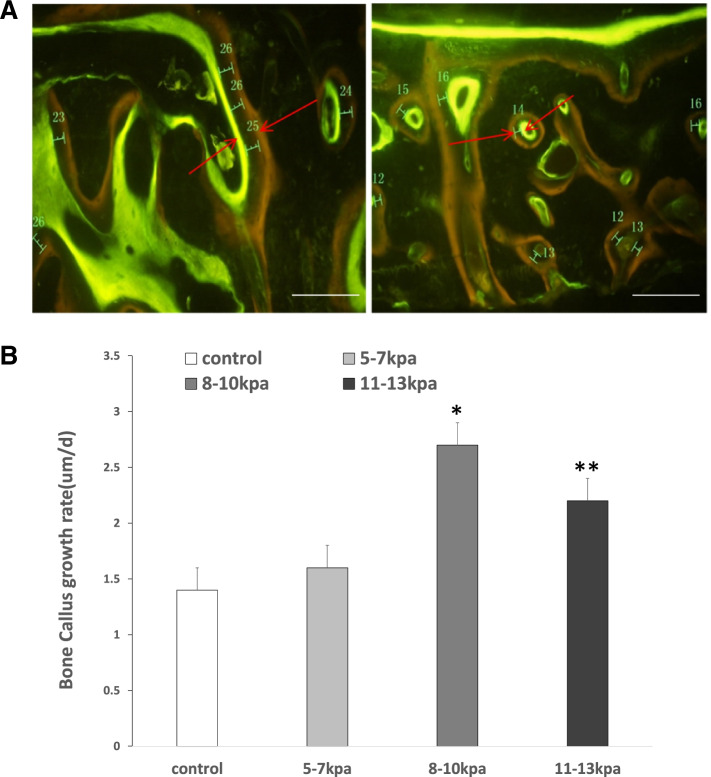
Fluorescent labeling was detected in newly formed bone tissue 4 weeks after surgery. Notes: **A** Images of fluorescence double staining for tetracycline (yellow) and calcein (green) in the defects in different groups; scale bars: 100 μm. **B** Mineral apposition rates for different groups. Results are mean ± standard deviation (χ ± SD); *P < 0.05 vs group **A**, **B**, **D**; **P < 0.05 vs group **A**, **B**

Fluorescent labeling was detected in newly formed bone tissue at the fourth week (Fig. 3). The two images were both taken with an optical microscope at a magnification of 200. The results showed the speed of new bone formation in Group C was 2.6 ± 0.3 µm/day and faster than that in other groups, among which the control group had the slowest speed of new bone formation. The difference was statistically significant (*P* = 0.005).

### Histology analysis

**Fig. 4:**
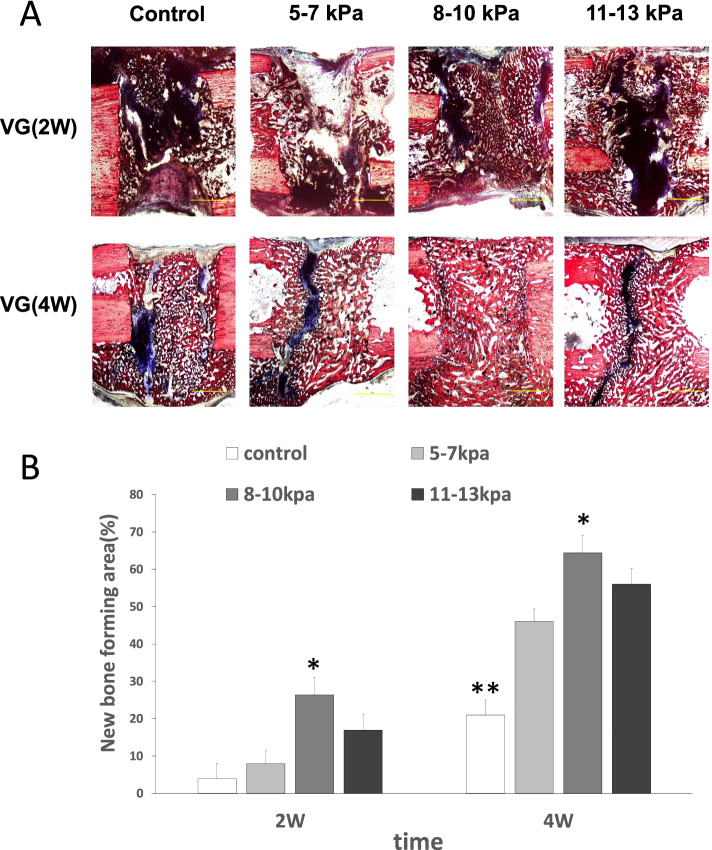
VG staining at the 2 and the 4 weeks after surgery. Notes: **A** The newly formed bone is stained red, fibrous tissue is blue and the defect are black (Van Gieson’s staining); scale bars: 2 mm. **B** The percentage of bone-forming area for different groups. Results are mean ± standard deviation (χ ± SD); *P < 0.05 vs group **A**, **B**, **D**; **P < 0.05 vs group **B**, **C**, **D**

Two weeks after surgery, no apparent bone tissue but a little fibrous tissue and cartilage tissue were easily detected by light microscopy in groups A and B. Growing woven bone and more cartilage tissue appeared around the defect sites in group C and D. Four weeks after surgery, the growing woven bone and cartilage tissue filled the bone defect sites but the gap was still obvious in group A, B and D. However, the woven bone fully bridged the defect in group C and completely filled the defect (Fig. 4). The percentage of bone-forming area in group C was 26.36 ± 4.16% at the second week and 64.37 ± 3.61% at the fourth week, which was significantly superior to other groups (*P* < 0.05).

## Discussion

In previous studies, intermittent pneumatic soft-tissue compression (IPC) has been proved to be effective for bone fracture healing by enhancing osteogenesis and vascularization. Until now, no study has investigated the effect of optimal intensity of IPC on bone defects, limiting the clinical application of treating the bone defect patients. In our study, optimal intensity of intermittent pneumatic soft-tissue compression on bone defect repair was determined. Our results showed that the beneficial effects of IPC were affected by the treatment intensity. The findings may help optimize an IPC treatment protocol for bone defect repair.

Park reported the effect of sequential pneumatic compression on a 3 mm ostectomy created in rabbit tibiae [10]. The use of sequential compression showed increased bone formation in the experimental group compared with the controls. Unlike our study, they set a much shorter inflation time of 2 s followed by a deflation period of 20 s and treated their subjects for 1 h per day for 25 days. The difference in treatment regimens made it difficult to determine which variables may have contributed to the enhanced bone healing, and it was essential to optimize the IPC treatment protocol for clinical use. In our study, we chose the 15 Hz frequency of compression and the stimulation lasted 30 min every day. This situation could be more controlled and decreased the interference of treatment regimens. In clinical practice, patients with bone defects or bone fractures commonly get fixation by steel plate or external fixation to prevent bone movement. And then the intermittent pneumatic compression can be used in the upper proximal to the defect site and used an immobilization to prevent bone movement. This therapeutic will not get extra pain in patients.

The mechanism of IPC increasing the bone regeneration is still unclear. Possible mechanisms include an increase in, or a physical or chemical effect on the vascularity at the fracture site. Other possibilities could be altered mechanical forces at the fracture callous or on the cortical or cancellous bone. A few studies suggested that IPC can increase the flow of interstitial fluid across bone tissue and this may play an important role in bone tissue metabolism [17–19]. In natural bone tissue, interstitial fluid flows from the medullary venous sinusoids across cortical bone to the periosteal lymphatics. IPC can increase the flow of interstitial and venous pressure, and the changes of interstitial flow may enhance osteoblast function through various mechanisms such as streaming potentials, fluid shear stress, or mechanical strain induced by fluid flow [20, 21]. Similarly, increased fluid flow also can improve the transport of nutrients to the new bone tissue formed in repair process [22]. Several studies revealed that IPC may produce positive stresses on endothelium and osteoblast [18, 19]. More research will need to focus on the mechanism of IPC increasing the bone regeneration.

Our study has limitations in its design and application. In our experiment, we may not have allowed sufficient time for new bone formation to fill the defect. After the woven bone fully bridged the defect and completely filled the defect in group C 4 weeks after surgery, we stopped the observation. At this time point, the results showed the significant superiority in optimal treatment group. After longer time point, the 5 mm radial bone defect in rabbits will be completely filled and fully bridged with or without any treatment. So it may be difficult to distinguish the difference between each group in longer observation period. However, more time may be needed to contribute to better bone formation and mechanical strength. Additionally, the stimulation lasted 30 min every day and the frequency of compression was 15 Hz tested in our study. Other duration or frequency may get a different effect and requires further study. Finally, although increased bone formation was observed in the animal model and the optimal intensity was 8–10 kPa, there are significant diversities in human application. The optimal osteogenic effect of the compression device for human patients warrants further research.

Overall, our study showed that the beneficial effect of IPC on the bone defect was affected by the treatment intensity. Radiographic and histologic evidence has been shown that IPC can enhance bone tissue formation and optimal intensity is 8–10 kPa for repairing the rabbit radial bone defect. Although the optimal osteogenic effect of the compression device for human patients warrants further evaluation, a therapeutic technique for enhancing bone formation could be widely applicable to the treatment of bone defects.

## Data Availability

The datasets generated and/or analyzed during the current study are included within the article. The underlying materials are available from the corresponding author on reasonable request.

## Abbreviations

BMP-2: Bone morphogenetic protein-2
VEGF: Vascular endothelial growth factor
IPC: Intermittent pneumatic compression
DVT: Deep vein thrombosis
Micro-CT: Micro-computerized tomography
Tb.N: Trabecular number
Tb.Sp: Trabecular spacing
BMD: Bone mineral density
MMA: Methyl methacrylate
